# Demographic and clinical predictors of depressive symptoms among incarcerated women

**DOI:** 10.1186/1744-859X-9-34

**Published:** 2010-09-06

**Authors:** Carmen SV Pinese, Antonia RF Furegato, Jair LF Santos

**Affiliations:** 1 Department of Psychiatric Nursing and Human Sciences, College of Nursing, University of São Paulo at Ribeirão Preto, Ribeirão Preto, Brazil

## Abstract

**Background:**

Imprisonment may lead to the development of mental illness, especially depression. This study examines the clinical and sociodemographic profiles of imprisoned women, identifies indicative signs of depression, and relates these indicators to other variables.

**Methods:**

This study took the form of descriptive exploratory research with a psychometric evaluation. A total of 100 of 300 women in a female penitentiary were interviewed. A questionnaire with sociodemographic, clinical and penal situation information was used, along with the Beck Depression Inventory. The authors performed bivariate and multivariate analysis regarding depression.

**Results:**

In all, 82 women presented signs of depression (light = 33, mild = 29 and severe = 20). Comorbidities, lack of religious practice, absence of visitors and presence of eating disorders were risk factors for depression (*P *= 0.03, 0.03, 0.02, 0.04, and 0.01). Being older was a protection factor against severe depression; for women over 30, the risk of depression was multiplied by 0.12. The rate of depression among women prisoners was high.

**Conclusions:**

Comorbidities, the lack of religious practice, not having visitors and eating disorders are significant risk factors for depression, while age is a protective factor, among incarcerated women.

## Introduction

The prison population, especially the female sector, grows every day. Data from the Brazilian National Penitentiary Department shows that in 2005 the prison system population in the State of São Paulo was 120,601 with 3,903 women, and in 2008, it was 145,096 with 6,520 women. Among the reasons that result in women being sent to prison is involvement with drug users/traffickers [1,2].

Epidemiological studies have shown gender differences in the occurrence, prevalence and course of mental behavior issues and disorders. Women present exceptional vulnerability to symptoms of depression and anxiety, mainly associated with their reproductive period [3]. The prevalence ratio for women has varied from 1.5 to 3.0, reaching an average female to male ratio of 2:1 [4-7].

The prison environment neutralizes the formation and development of basic human values, contributing to stigmatization, altering the convict's conduct and leading to temporary or even irreversible psychic sequelae [8,9].

Mental disorders occur frequently in the context of reclusion. Although evidence suggests that imprisonment conditions can lead to anxiety, depression, self-harming or heteroaggressive behavior, obsessions, psychoactive substance abuse and suicide, there is no agreement in the literature on the causal relationship between confinement and mental disorders [8,10-12].

In addition to feelings of inadequacy, important feelings in imprisoned people are anticipated suffering in life outside of incarceration, fear of family abandonment, guilt for being absent from raising and educating their children, losing their right to the social importance of work, identity loss, social discrimination that impairs prospects for working outside of the criminal context, and social recognition [12-14]. This study was carried out due to the high frequency of depression among women inmates with the belief that these women need improved conditions to be able to serve their time with dignity.

This study identifies the clinical, sociodemographic and penal profile of women in a prison unit of the State of São Paulo, and evaluates possible associations of these variables to indicators of depression.

## Methods

This is an analytic descriptive study using psychometric evaluation. The research was carried out in the Health Clinic of a female penitentiary in Ribeirão Preto, Brazil. The institution has a holding capacity of 310 women. Those under a disciplinary regime were excluded (around 10 women). A disciplinary regime involves women that have broken the rules of the institution and have been removed from the main prison population for a period of time, depending on the severity of the infraction, and according to what is proscribed in the Law for Penal Execution.

Among the 300 inmates, 100 took part in the study (33% of the viable population). At first, the selection criterion for the sample would be to choose the 100 women that had just come into the Prison Unit. After a pilot test, the identification instrument was adapted. The authors decided to randomly select participants by drafting 100 inmates, regardless of the time spent in the prison unit. In this way, the same inclusion probabilities were given to all inmates.

### Clinical, sociodemographic and penal questionnaire

The questionnaire used is a descriptive instrument comprised of questions pertaining to identification and sociodemographic data, including clinical information and penal situation. The subject's identification starts with a code (no names were used), with information regarding confinement start date in the institution, age, ethnicity, marital status (married, non-married), education level, profession, work, origin, place of living, family income, religion, and included a visitors log. Clinical information was evaluated for the presence of diseases or illnesses, disabilities, limitations, medication use, number of children and abortions, weight, alteration of eating habits, physical status, sexual activity, and smoking habits. The penal situation survey topics were the article of the penal code violated, time sentenced and served and reconviction, if any.

The Beck Depression Inventory was used to assess depression. It evaluates the presence of depressive symptoms, with 21 items, using 4 levels of intensity. The Beck Depression Inventory [15] has been translated and validated in Portuguese [16,17], with a Cronbach α of 0.81 and mean score of 8.5 ± 7.0, similar to several other studies.

Data were collected between May and September 2007. Subjects were interviewed individually, and the researcher filled out the answer forms at the time in order to minimize loss of information.

The project was approved by the Ethics on Research Committee of the EERP-USP (Proc. 0687), and by the directors of the Female Penitentiary of Ribeirão Preto. After explaining the goal of the interview, participation and use of results to the participants of this study, they signed free and informed consent forms. Two inmates did not agree to participate in the study, and so two additional inmates were selected from the population.

Cases identified as positive for depression were forwarded to professionals involved in medical and nursing care within the institution. The most severe cases were forwarded to specialized psychiatric care.

After statistical and descriptive data analysis, depression was related to the other variables through multivariate and bivariate analysis. The independent variables used were: age, ethnicity, living together with a partner, education level, comorbidities, religion, eating habits, visitors, sleep, and tobacco use. All were treated as binomials with values of 0 and 1.

Dependent variable depression was classified into four levels: no depression, light, mild and severe depression, using the Beck scale. Due to the high occurrence of people with symptoms suggesting severe depression, including those presenting suicide risks, cut-off values for the suspected population were adopted and defined as: absence of depression (0-9), light depression/dysthymia (10-18), mild depression (19-29), severe depression (30 or more), as suggested by Beck *et al*. [18].

The multinomial logistic regression model was adopted with the outcome variables having the classes 0, 1, 2 or 3 in order to perform a compound appreciation of the possible influences of the independent variables on depression. The relative risk ratio was chosen as the comparison element [19]. Factors associated with each category of depression were compared to the base category (no depression) and *P *values smaller than 0.05 were considered significant. The discussion of the results is supported by theoretical references from the literature on the theme.

## Results

### Clinical, sociodemographic and penal profile

#### Subject identification

The 100 women interviewed ranged in age from 20 to 63, most of whom were between 20 and 29 (52%), Caucasian (65%), and single (70%); 45% noted having partners (Table 1).

**Table 1:** Inmate distribution in a female penitentiary according to their sociodemographic features (n = 100)

Sociodemographic feature		Value
Age	20-30	52
	30-40	28
	40+	20
Ethnicity	Caucasian	65
	Black	35
Origin	City of Ribeirão Preto	4
	Region	5
	Other regions of the state	80
	Other state	10
	Other country	1
Marital status	Single	70
	Married	15
	Separated	11
	Widowed	4
Education	Illiterate	3
	Incomplete fundamental education	49
	Complete fundamental education	7
	Incomplete high school	21
	Complete high school	14
	Incomplete university degree	4
	University degree	2

Only three were illiterate, 77% had elementary education or had not completed high school, 20% had completed high school and two had college degrees. Most of them had worked before being incarcerated (62%). Family monthly income varied from up to minimum wage (29%), between one and two times minimum wage (26%), between two and three times minimum wage (14%), and above three times minimum wage (31%). The Brazilian minimum wage is about US $250/month.

For housing, 61% owned their own houses, 26% rented and 13% borrowed. Eight of them lived alone and 82% ranged from two to seven people living in the same house.

For religion, 45% were Catholics, 37% were part of the Evangelical church, and 57% of this total practiced their religion.

In all, 4 women were from the city of Ribeirão Preto and 5 were from the region; 80 women were from other regions of the state, 10 from other states, and 1 from another country.

#### Clinical information

According to the self-evaluation of the studied women, almost 50% had no diseases; 25% had cardiorespiratory diseases and hypertension, 6% had psychiatric disorders and 19% had other diseases. The majority did not present any kind of disability. Many women responded that their major limitation was being in prison. Authors also found eating and sleeping disorders, and a lack of physical and sexual activity; 60% of the women smoked (Table 2).

**Table 2:** Clinical information on 100 imprisoned women

Clinical information		%
Diseases	No diseases	45
	Cardiorespiratory	30
	Other	19
	Psychiatric	06
Disabilities	None	92
Obstetrics	No. of children:	
	None	21
	1-3	60
	≥4	19
	Abortion	29
	Active sexually	10
Eating habit alterations	Yes	32
Sleeping habit alterations	Yes	68
Practice physical activity	Yes	23
Smoking	No. of cigarettes/day:	
	None	42
	Less than 19	24
	More than 20	34

The tests indicated a significant prevalence of depression among women in prison, although they do not recognize it in their self-evaluation. The cases identified were managed and referred for specialized treatment.

### Penal situation

The most frequently violated Brazilian penal code among the inmates was penal code 12: Illicit drugs trafficking (64%). Conviction time varied from no current conviction (that is, awaiting trial = 11) to 3 years (18). Most of them had already completed 2 years of their sentence (58%) or 2 to 4 years (32%).

### Depression and the variables

The results demonstrated that among the 100 women interviewed, 82 presented indicative signs of depression, and 20 of them were considered severe. Objective data and the relationship with depression are shown in Table 3, highlighting the higher frequencies of light, mild and severe depression. Complementary data, mainly subjective data, are presented below.

**Table 3:** Independent variable frequencies by levels of depression and *P *value of Mann-Whitney test

Variables	*P *value	Category	N	Depression levels							
				
				No depression		Light		Mild		Severe	
				
				N	%	N	%	N	%	N	%
Age	0.206	Under 29	52	08	15.4	15	29	19	36.5	10	19.1
		≥30	48	10	20.8	18	37.6	10	20.8	10	20.8
Ethnicity	0.704	Caucasian	65	13	20	19	29.3	18	27.7	15	23
		Non-Caucasian	35	5	14.3	14	40	11	31.4	5	14.3
Educational level	0.492	Up to complete fundamental education	59	10	17	20	34	14	23.7	15	25.3
		Above	41	8	19.5	13	31.7	15	36.6	5	12.2
Eating Habits	0.000	No alterations	47	13	27.7	20	42.5	10	21.3	4	8.5
		Alterations	53	5	9.4	13	24.5	19	35.9	16	30.2
Sleep	0.000	No alterations	68	18	26.5	22	32.4	19	27.9	9	13.2
		Alterations	32			11	34.4	10	31.2	11	34.4
Tobacco	0.290	No	42	5	12	21	50	12	28.5	04	9.5
		Yes	58	13	22.4	12	20.7	17	29.3	16	27.6
Comorbidities	0.155	No	45	11	24.5	14	31.1	15	33.3	5	11.1
		Yes	55	7	12.7	19	34.5	14	25.5	15	27.3
Religion	0.810	Practicing	57	12	21	20	35	12	21	13	23
		Not practicing	42	6	24.3	13	32	16	38	7	16.7
Marital status	0.358	Married	45	8	17.8	11	24.5	18	40	8	17.7
		No married	55	10	18.2	22	40	11	20	12	21.8
Visitors	0.322	Yes	49	11	22.5	14	28.6	18	36.7	6	12.2
		No	51	7	13.7	19	37.3	11	21.6	14	27.4
Total			100	18		33		29		20	

Of the eight women living alone, only one of them showed no signs of depression. The most severe cases of depression were among Catholics and women from the Evangelical church. Of the 20 severe depression cases, 3 had had 1 abortion and 1 had had more than 5 abortions.

Eating habit alterations showed significant results regarding depression. All 18 women with no signs of depression reported that they did not have sleeping pattern alterations. As for the 20 severe cases of depression, 9 reported that they did not have sleeping pattern alterations.

Although the prison offered physical activity programs, 77% of women did not take part in them. Even when showing no depression, most women did not practice physical activity. Among the 18 women that showed no signs of depression were all the women who had worked before imprisonment.

Of the 89 convicted women, 72 showed signs of depression. As for reconviction, 81 women were first-time offenders. Of the 20 women showing signs of severe depression, 5 were reconvicted. In one case this was for the seventh time.

In the multivariate analysis, age, comorbidities, religion, eating habit alterations and receiving visitors were significant variables (Table 4). The presence of comorbidities multiplied the risk for light depression by a factor of 5.43 and for severe depression by 8.81. Not practicing religion increased the probability of presenting mild depression (6.09). Eating habit disorders were strongly associated with mild depression (5.7) and with severe depression (11.11). A strong association between not receiving visitors and showing severe depression was shown (9.15). The variable sleep was excluded from the regression analysis because of instability: the contrast category (yes) has a null frequency scale.

**Table 4:** Multinomial logistic regression for the outcome variable 'depression' among inmates

Independent variables	Outcome variable								
	
	Light depression			Mild depression			Severe depression		
	Relative risk ratio	*P *value	Standard error	Relative risk ratio	*P *value	Standard error	Relative risk ratio	*P *value	Standard error
Age	0.47	0.34	0.37	0.23	0.08	0.19	0.12	0.04	0.12
Ethnicity	2.00	0.39	1.60	1.21	0.82	1.00	0.51	0.502	0.51
Marital status	1.27	0.76	0.97	0.39	0.24	0.31	1.89	0.504	1.81
Education	0.51	0.37	0.38	0.93	0.93	0.73	0.17	0.09	0.17
Comorbidities	5.43	**0.04**	4.38	3.17	0.17	2.66	8.81	**0.03**	8.80
Religion	2.76	0.21	2.25	6.09	**0.03**	0.60	5.78	0.08	5.80
Eating habits	1.46	0.63	1.16	5.70	**0.04**	4.71	11.11	**0.01**	10.93
Visitors	2.57	0.21	1.92	2.23	0.32	1.78	9.15	**0.02**	8.66
Tobacco	0.22	0.06	0.17	0.70	0.68	0.60	2.13	0.453	2.15

Wald statistics = w = 77.7 *P *< 0.000 Pseudo RZ = 0.289. Bold types for p values indicate significance at the level of 0.05.

Being older was a protecting factor for severe depression. In other words, women over 30 present the risk of being in this category multiplied by a factor of 0.12.

## Discussion

In typical mild, moderate, or severe depressive episodes, the patient suffers from a lowering of mood, reduction of energy, and decrease in activity. Capacity for enjoyment, interest, and concentration are reduced, and marked tiredness after even minimum effort is common. Sleep is usually disturbed and appetite diminished. Self-esteem and self-confidence are almost always reduced and, even in the mild form, some ideas of guilt or worthlessness are often present. The lowered mood varies little from day to day, is unresponsive to circumstances and may be accompanied by so-called 'somatic' symptoms, such as loss of interest and pleasurable feelings, waking in the morning several hours before the usual time, depression worsening in the morning, marked psychomotor retardation, agitation, loss of appetite, weight loss, and loss of libido [20]. The subsyndromic expressions of depressive disorders are more difficult to study, but also have a significant negative impact on patients' quality of life [6,15,21].

Data on the presence of depression among convicted women in this study are reason for concern. They are above the general population indexes [4,6,7,14]. Data in the literature diverge: in one report, between one-third and one-half of the British female penitentiary population presented some type of mental disorder [10]. In Chicago, mental disorder rates in the imprisoned population were three to four times higher than the general population; these rates were even higher when only women were considered [11]. Further, a Brazilian study demonstrated a low prevalence of psychiatric cases among the female prison population [12].

In the 40 years old or above age group, this study demonstrated a higher percentage of women showing no signs of depression. The multivariate analysis showed that being in the age group 30 years old or more is a protective factor for depression. This finding contrasts with what is known about the association between old age and depression, but it must be noted that 30 years is a very low cut off for age.

Although 70 inmates were single, 45 reported having a companion. A fact to note is that of the 20 women with severe depression, 16 were single. In contrast, mild depression was more frequent among those that reported having a partner. Being married is associated with a lower rate of depression in men; however, being single is a condition associated with a lower rate of depression in women, as found in gender specific studies [21,22].

Both in this study and in a study carried out in a female penitentiary in Rio de Janeiro, there was no direct relationship between low education and socioeconomic condition with criminal rates [23] and depression. Neither having a job previous to imprisonment nor working as an option in the institution showed significant differences regarding depression. Most of the inmates did not take part in any type of activity, particularly those with severe depression. Being depressed acts as an inhibiting factor of the will, initiative for practicing physical activities and other efforts. In contrast, working and exercise could stimulate positive attitudes in these women [13].

A total of 12 women reported not having any religion and, within this group, no case of severe depression was found. However, when the variable was submitted to multivariate analysis for mild depression, it demonstrated a strong relationship between having a religion and developing mild depression. Another study has demonstrated that religion can ease the routine and burden of convicted women in prison [12].

Depression is an illness that frequently accompanies comorbidities, especially chronic diseases and alcohol and drug use. Prevalence rates for depressive disorders among somatic disease patients are substantial, from 22 to 33%. This is frequently a source of difficulty in the diagnoses of depression in primary health services [6,24].

Among the inmates surveyed in this study, of the 23 that presented cardiac and respiratory diseases, 22 showed signs of depression. The most common general comorbidities were high blood pressure, ischemic diseases, hypothyroidism, other thyroid disorders, and diabetes. The fact that comorbidities were highly significant for light and severe depression stands out as confirming the data in the literature [25,26].

Many inmates affirm not having any diseases, disabilities or limitations. However, among eight people that reported some type of disability (five with visual disabilities, one with a mobility disability and dwarfism) all had some degree of depression, except one that presented stroke sequelae with no signs of depression. Regarding limitations, apart from being imprisoned (with no right to leave), most women feel they have no limitations. Although rates of women with disabilities and limitations were the same, they did not overlap. Some women that objectively had disabilities subjectively did not feel they had any limitations.

Eating habit alterations showed significant results in the multivariate analysis of this study regarding depression. Of all 82 cases showing depression, 53 reported eating habit alterations, confirming the data in the literature on this subject [24]. Sleeping habit alterations were also noted. Many women reported that they wake up during their sleep. Depression is characterized by frequently disturbed sleep, usually by terminal insomnia. Decrease in appetite is also present, generally followed by a slight weight loss [27,28].

Regarding the obstetrical aspects surveyed in this study, of the 60 women that had 1-3 children, 50 showed some degree of depression, and among these 13 showed severe depression. A study carried out on women aged between 45 and 55 years old in Poland found more pregnancies, more abortions, pregnancy complications, and post-labor depression in women with higher levels of depression. A study carried out in a female police station in Belo Horizonte pointed to a relationship between starting a criminal life and maternity. Women justify crimes by trying to ensure comfort and the acquisition of consumer goods for their children [29].

Receiving visitors was a relevant factor for women with severe depression. When they are incarcerated, women are also forced into separation from their children and family, causing grief, distress, loneliness, longing, loss, and regret. In addition, intimate visits are difficult [1]. However, family, religious and professional links can act as positive supports to depressive persons.

Of the 100 women interviewed, many smoked more than 20 cigarettes a day and 11 showed signs of severe depression. Despite these indicators, the multivariate analysis did not find any direct relationship between smoking and depression. The literature affirms that being a smoker can increase the frequency of a depressed state [6,14,24].

The major criminal offenses were drug trafficking (64%) and robbery (12%); 81% were in prison for the first time, 89% had already being convicted and 59% of them had been in prison for at least 2 years [2]. When analyzed by nursing staff in the context of a female prison, the nurse does not see the crime committed by the inmate, but only the consequences to her rehabilitation [30].

It is important to point out that in spite of the fact that some variables are totally independent from the outcome variable (depressive symptom), the results confirm their connection to the depressive clinical status.

This study did not aim to investigate the 'possible' use of illicit drugs in the prison system. However, the high rate of conviction for drug trafficking among the prisoners as well as the high frequency of depressive symptoms shows the need for further studies on this.

As this prison unit does not have specialized psychiatric care, the clinical diagnosis of depression was not carried out alongside the investigation. Suspect cases were forwarded for care and follow-up.

## Conclusions

Multivariate analysis has indicated the risk factors that contribute to the manifestation of depression (comorbidities, religion, eating habits and visitors), and that age can be a protective factor, for imprisoned women.

These results may provide information for planning special nursing care, and also the management of services and policies aimed at this population.

## Study limitations

The experience of carrying out this data collection in the field was enriching and, at the same time, wearying. The theme of the research is profound, causing deep emotional responses in the inmates interviewed. The nurse spent the necessary time to listen to the inmates fully, a fact that consequently reduced the number of data collections possible per day.

## Competing interests

The authors declare that they have no competing interests.

## Authors' contributions

CSVP and ARFF conceived this study, and participated in its design and coordination. CSVP did the data collection. JLFS participated in the design of the study and performed the statistical analysis. All authors read and approved the final manuscript.
